# Fabrication of Polyurethane Elastomer/Hindered Phenol Composites with Tunable Damping Property

**DOI:** 10.3390/ijms24054662

**Published:** 2023-02-28

**Authors:** Xiuying Zhao, Ruiheng Jin, Zhihao Niu, Yangyang Gao, Shikai Hu

**Affiliations:** 1 State Key Laboratory of Organic-Inorganic Composites, Beijing University of Chemical Technology, Beijing 100029, China; 2 Key Laboratory of Carbon Fiber and Functional Polymers, Ministry of Education, Beijing University of Chemical Technology, Beijing 100029, China; 3 Beijing Engineering Research Center of Advanced Elastomers, Beijing University of Chemical Technology, Beijing 100029, China

**Keywords:** damping property, hindered phenol, polyurethane, composites

## Abstract

Vibration and noise-reduction materials are indispensable in various fields. Polyurethane (PU)-based damping materials can dissipate the external mechanical and acoustic energy through molecular chain movements to mitigate the adverse effects of vibrations and noise. In this study, PU-based damping composites were obtained by compositing PU rubber prepared using 3-methyltetrahydrofuran/tetrahydrofuran copolyether glycol, 4,4′-diphenylmethane diisocyanate, and trimethylolpropane monoallyl ether as raw materials with hindered phenol, viz., and 3,9-bis{2-[3-(3-*tert*-butyl-4-hydroxy-5-methylphenyl)proponyloxy]-1,1-dimethylethyl}-2,4,8,10-tetraoxaspiro[5.5]undecane (AO-80). Fourier transform infrared spectroscopy, thermogravimetric analysis, differential scanning calorimetry, dynamic mechanical analysis, and tensile tests were conducted to evaluate the properties of the resulting composites. The glass transition temperature of the composite increased from −40 to −23 °C, and the tan *δ*_Max_ of the PU rubber increased by 81%, from 0.86 to 1.56 when 30 phr of AO-80 was added. This study provides a new platform for the design and preparation of damping materials for industrial applications and daily life.

## 1. Introduction

Noise and vibration can have adverse effects on heavy machinery [1], buildings and structures [2], military equipment [3], and human physical and mental health [4,5,6,7], among others. Therefore, vibration- and noise-reduction materials are widely employed as indispensable functional materials. Polymers are typically used as damping materials to absorb noise and reduce vibrations [8]. When acoustic waves from vibrations or noise are transferred to polymeric materials, the delivered mechanical and acoustic energies are converted to molecular chain movements, resulting in energy dissipation through internal friction between the molecular chains [9,10]. Among polymeric materials, rubber materials possess excellent damping and vibration-reduction properties,5 owing to their unique viscoelasticity [11].

The damping property of polyurethane (PU) can be flexibly modulated according to the application environment, owing to the strong designability of its molecular structure [12,13,14,15,16,17,18,19]. Polyester-based PU is easily hydrolyzable and unstable, whereas polyether-based PU, which is mainly composed of ether bonds, exhibits excellent resistance to hydrolysis [20]. Poly(tetramethylene ether)glycol is widely used to synthesize PU; however, its strong tendency to crystallize can affect the elasticity of PU [21,22,23]. Modification, blending, copolymerization, and interpenetration of polymer networks can be conducted to improve the damping properties of polymers [24,25,26,27,28]. Millable PU (MPU) contains unsaturated double bonds that can serve as crosslinking sites for reactions with a vulcanizing agent [29]. After processing with mixing equipment, raw MPU rubber can be vulcanized and shaped using various molds. In addition to designing the molecular structure of PU, the damping property of MPU can be modulated by designing the vulcanized formula [30,31].

As a type of polyether, soft segments with low crystallinity at room temperature, 3-methyltetrahydrofuran/tetrahydrofuran copolyether glycol (3MCPG) possesses excellent low-temperature flexibility [32]. Owing to its side-chain methyl groups, the friction between the molecular chains of 3MCPG increases during movement, which significantly improves the damping property of PU rubber [12,33,34]. PU synthesized using 3MCPG as a soft segment is a polymeric material with great developmental potential.

Hindered phenols can form hydrogen bonds with polar polymer matrices [31,35]. Under an external force, the mechanical and acoustic energies are constantly converted to thermal energy via the destruction and reconstruction of the intermolecular hydrogen bonds in the polymer matrix, resulting in significantly improved damping properties of the polymeric material [36,37,38,39]. Therefore, Hindered phenols are widely used to improve the damping property of polymers [35,40,41].

In this work, 3MCPG, 4,4′-diphenylmethane diisocyanate (MDI), and trimethylolpropane monoallyl ether (TME) were used as raw materials to synthesize MPU. Next, a small-molecular hindered phenol, namely, 3,9-bis {2-[3-(3-*tert*-butyl-4-hydroxy-5-methylphenyl)propionyloxy]-1,1-dimethyl}-2,4,8,10-tetraoxaspiro[5.5]undecane (AO-80), was added to MPU to obtain a series of MPU/AO-80 composites. Systematic analyses of the material properties of the composites revealed that the MPU/AO-80 composites possessed an excellent damping property. The tan *δ* of the composite with 32 phr of AO-80 increased by 81% from 0.86 to 1.56. This study provides a novel route for the development of new polymeric materials with excellent damping properties.

## 2. Results and Discussion

### 2.1. Preparation of Raw MPU Rubber

**Figure 1:**
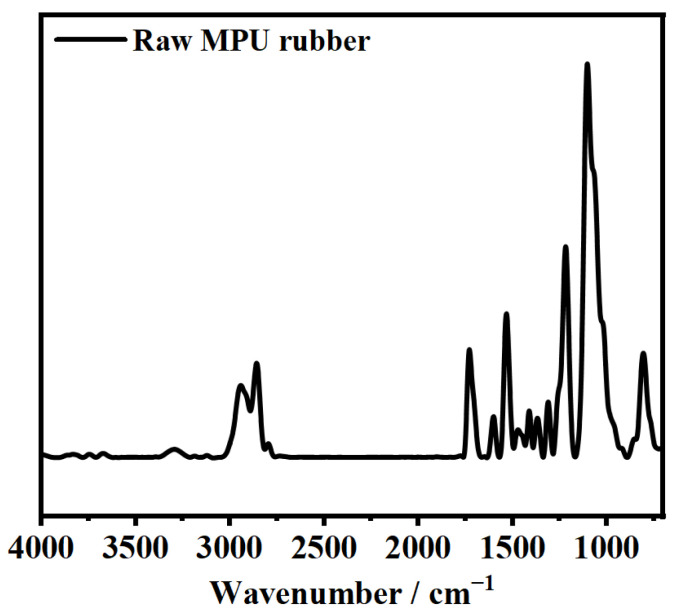
FTIR spectrum of raw MPU rubber.

**Figure 2:**
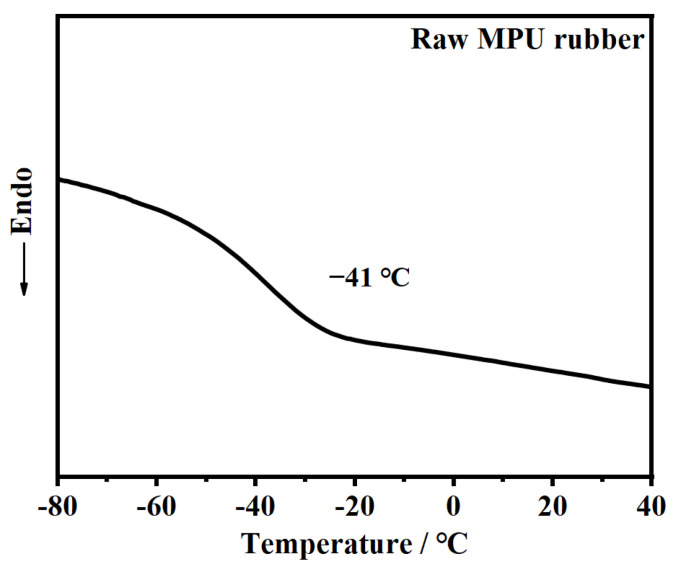
DSC curve of raw MPU rubber.

The chemical structure of the raw MPU rubber was studied using FTIR spectroscopy. The FTIR spectrum of the raw MPU rubber in Figure 1 shows a strong absorption peak of the ether groups (–O–) near 1100 cm^–1^, a broad absorption peak of the hydrocarbon groups at ~2900 cm^–1^, and an absorption peak of the imino group (N–H) at ~3300 cm^−1^. No absorption peak of the isocyanate group (–NCO) appeared near 2300 cm^−1^, confirming the successful synthesis of raw MPU rubber. The glass transition temperature (*T*_g_) of raw MPU rubber was determined to be −41 °C by DSC (see Figure 2).

**Figure 3:**
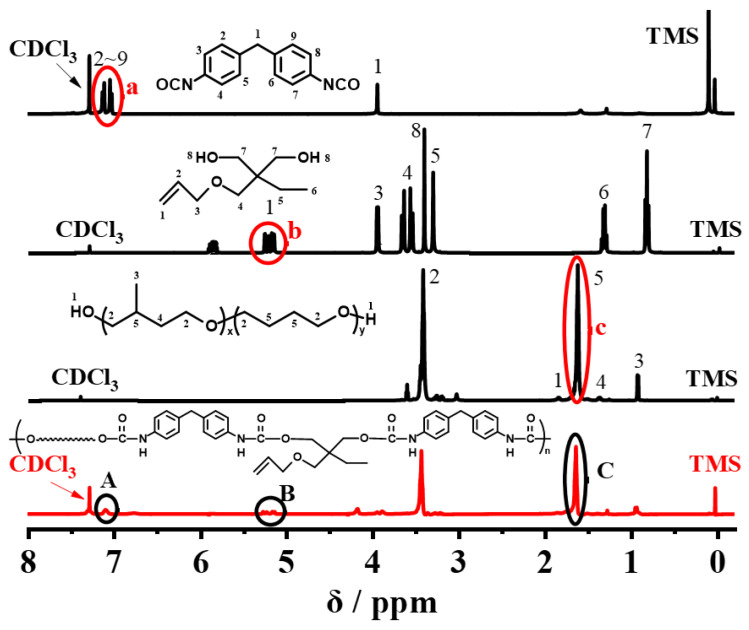
^1^H NMR spectra of 3MCPG, MDI, TME, and raw MPU rubber.

The ^1^H NMR spectra of 3MCPG, MDI, TME, and raw MPU rubber are shown in Figure 3. Peak a (δ = 7.01–7.19 ppm) in the ^1^H NMR spectrum of MDI corresponds to its benzene ring protons. Peak b (δ = 5.10–5.27 ppm; –CH=) in the spectrum of TME corresponds to the protons belonging to its double bond, and peak c (δ = 1.57–1.66 ppm; –CH_2_–) in the spectrum of 3MCPG corresponds to the protons on the methylene group not connected to the oxygen atom in the 3MCPG molecular chain.

Furthermore, in the ^1^H NMR spectrum of raw MPU rubber, peak A (δ = 7.03–7.14 ppm) corresponds to the protons on the benzene ring of raw MPU rubber. Several other characteristic peaks overlap with the solvent (CDCl_3_) peak. Peak B (δ = 5.12–5.28 ppm; –CH=) corresponds to the protons of the C=C double bond in raw MPU rubber, and peak C (δ = 1.60–1.71 ppm; –CH_2_–) corresponds to the protons on the methylene group not connected to the oxygen atom in the molecular chain of 3MCPG. These results confirm that unsaturated double bonds were successfully introduced into the macromolecular chains of raw MPU.

### 2.2. Preparation and Molding of MPU/AO-80 Composites

**Figure 4:**
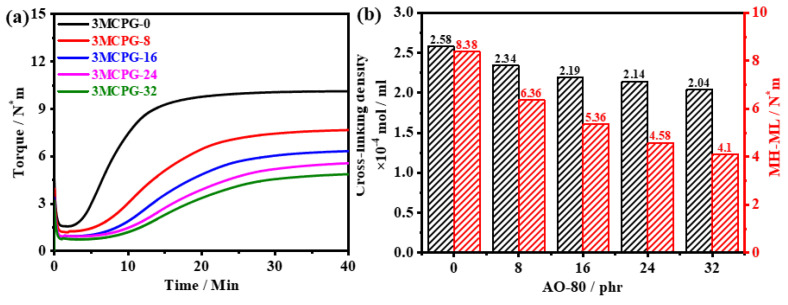
(**a**) Torque vs. melting time of the MPU/AO-80 composites. (**b**) Crosslinking densities and M_H_-M_L_ values of the MPU/AO-80 composites.

The vulcanization curves and crosslinking densities of the MPU/AO-80 composites with varying amounts of the AO-80 are shown in Figure 4a,b, respectively. As the AO-80 content increased, the maximum and minimum torque of the composite decreased; this is because AO-80 melts at 150 °C and acts as a plasticizer in the composite system. The torque difference and crosslinking density of the MPU/AO-80 composite decreased with increasing AO-80 content because free radical crosslinking occurs during the vulcanization of the MPU/AO-80 composite and the ability of AO-80 to scavenge free radicals affects the crosslinking process.

**Figure 5:**
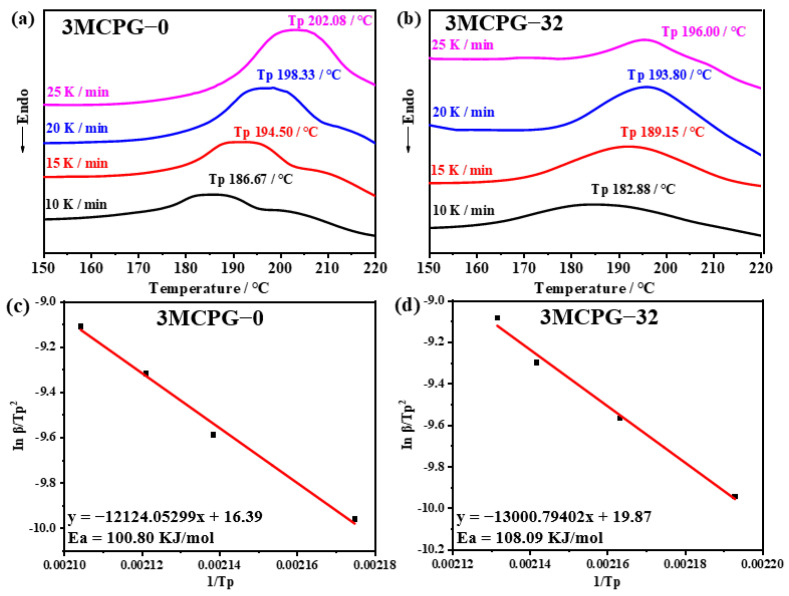
(**a**,**b**) DSC curves of (**a**) 3MCPG-0 and (**b**) 3MCPG-32 at different heating rates. (**c**,**d**) Kissinger equation fits for the DSC data of (**c**) 3MCPG-0 and (**d**) 3MCPG-32.

The DSC curves obtained at different heating rates for 3MCPG-0 (without the phenol additive) and 3MCPG-32 (with 32 phr of the phenol additive) are shown in Figure 5a,b, respectively. These curves clearly reveal that the exothermic peak shifts toward a higher temperature with an increased heating rate. At a given heating rate, the exothermic peak of the composite shifted to a lower temperature following the incorporation of AO-80. The Kissinger equation, shown in Equation (1), was used to calculate the apparent activation energy (*E*_a_) of the reaction [42,43].

(1) $$\ln\frac{\beta }{T_{p}{}^{2}}=\ln\frac{AR}{E_{a}}-\frac{E_{a}}{RT_{p}}$$

where *β* is the heating rate, *T*_p_ is the peak temperature, *E*_a_ is the reaction activation energy, and *R* (= 8.314510 J·mol^−1^·K^−1^) is the gas constant. The $\ln\frac{\beta }{T_{p}{}^{2}}$ vs.$\;\frac{1}{T_{p}}$ curves of 3MCPG-0 and 3MCPG-32 were fitted into Equation (1) to obtain linear fits, as shown in Figure 5c,d, and the extracted parameters are shown in Table 1. The slope of the straight line gives −*E*_a_/*R*. Thus, the apparent *E*_a_ of the mixtures can be calculated. The *E*_a_ values of 3MCPG-0 and 3MCPG-32 were determined to be 100.80 and 108.90 kJ/mol, respectively. These values indicate that the energy required for vulcanization increases with the addition of AO-80. Thus, AO-80 has a negative impact on the vulcanization process, which is consistent with the results obtained from the vulcanization curve and crosslinking density.

### 2.3. SEM of MPU/AO-80 Composites

**Figure 6:**
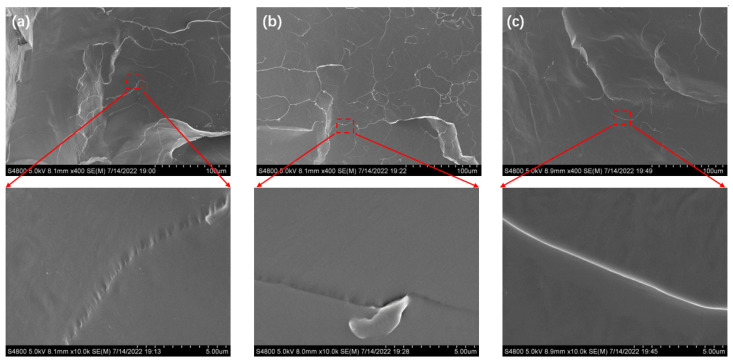
SEM images of the MPU/AO-80 composites: (**a**) 3MCPG-0, (**b**) 3MCPG-16, and (**c**) 3MCPG-32 (the lower panel is the magnification of the red-box part in the upper panel).

The micromorphologies of the MPU/AO-80 composites (3MCPG-0, 3MCPG-16, and 3MCPG-32) were studied through SEM to analyze the dispersion of AO-80 and the presence of vulcanization additives in the polymer matrix. For SEM, the cross-sections of the composites were quenched with liquid nitrogen prior to observation. As shown in Figure 6, similar to the quenched cross-section of the MPU matrix, the quenched cross-section of the MPU/AO-80 composite is extremely smooth and does not have any AO-80 aggregates. This is because the vulcanization molding was conducted at 150 °C and the peak melting temperature of AO-80 is 119 °C [44]. Thus, AO-80 melts into a liquid and is uniformly dispersed into the MPU matrix at high pressure. When the sample is cooled, no agglomerated AO-80 crystals appear.

**Figure 7:**
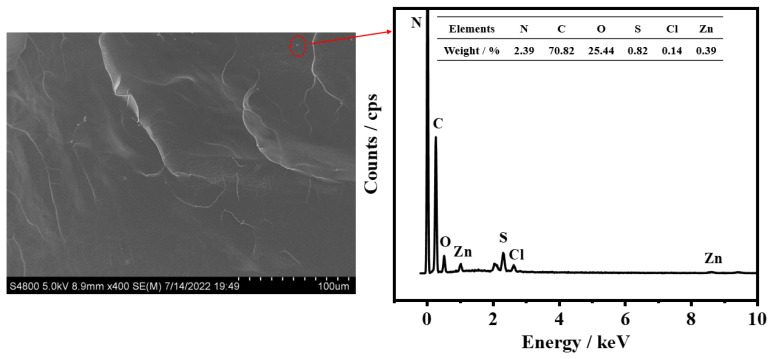
EDS profile of the MPU/AO-80 composite with 32 phr of AO-80 (3MCPG-32).

The SEM images of the composite samples (Figure 6) exhibit small granular particles, which were analyzed through energy-dispersive X-ray spectroscopy (EDS). As shown in Figure 7, the granular particles contained six elements, viz., N, C, O, S, Cl, and Zn, which belong to the active agents in NH-2 (the complex formed from the accelerator, dibenzothiazole disulfide, and zinc chloride).

### 2.4. TGA and DSC of MPU/AO-80 Composites

**Figure 8:**
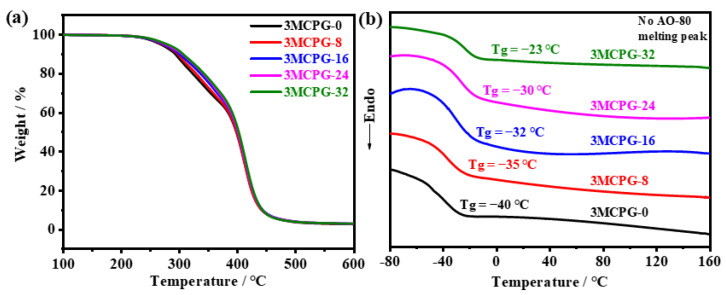
(**a**) TGA and (**b**) DSC curves of the MPU/AO-80 composites.

The TGA curves of the MPU/AO-80 composites are presented in Figure 8a. Table 2 provides detailed thermogravimetric data. As the amount of AO-80 added to the composites increased, the initial decomposition temperature (*T*_5%_) increased from 267 to 278 °C, the weight loss in the first stage decreased from 51.31 to 47.11%, and the residual mass increased from 2.66 to 2.93%. These results may be attributed to an increase in the number of intermolecular hydrogen bonds in the composite with increasing AO-80 content, which improves the thermal stability of the PU molecular chains to a certain extent [45].

As shown in Figure 8b, no melting peak of AO-80 was observed at 119 °C in the DSC curves of the MPU/AO-80 composites, confirming that AO-80 was dispersed well in the MPU matrix. The *T*_g_ of the composite increased with increasing AO-80 content, mainly because AO-80 forms intermolecular hydrogen bonds with the polar groups in MPU and these bonds not only restrict the movement of the polymer segments but also increase the energy required to initiate the movement of the molecular chains.

### 2.5. Static and Dynamic Mechanical Properties of MPU/AO-80 Composites

**Figure 9:**
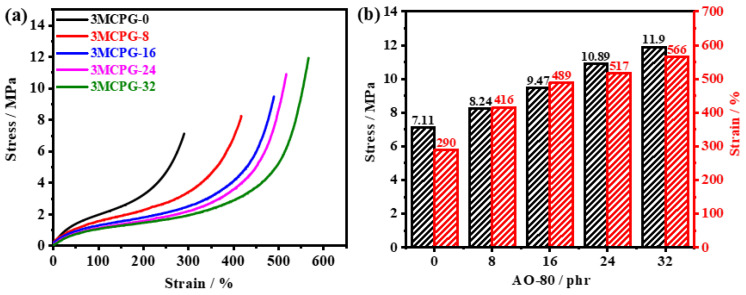
(**a**) Mechanical properties of the MPU/AO-80 composites. (**b**) Histogram of the mechanical parameters of the MPU/AO-80 composites.

The mechanical properties of the MPU/AO-80 composites were studied via tensile tests. The strain–stress curves are shown in Figure 9. As the AO-80 content increased, both the tensile strength and the elongation at the break of the MPU/AO-80 composites increased. This is because, when the elongation reaches a certain value, the hydrogen bonds formed between AO-80 and the MPU matrix break and the amount of AO-80 increases the number of hydrogen bonds formed between MPU and AO-80 [15]. The hydrogen bonds are re-formed in situ or ectopically so that the stress is redistributed and the molecular chains are in a relatively stable state. Owing to the reciprocal process of dynamic hydrogen-bond breakage and reconstruction processes, the ability of the material to dissipate external energy delivered to it is enhanced [46]. As the amount of AO-80 increased, the stress at 100% elongation decreased because the addition of AO-80 reduced the crosslinking density of the system.

**Figure 10:**
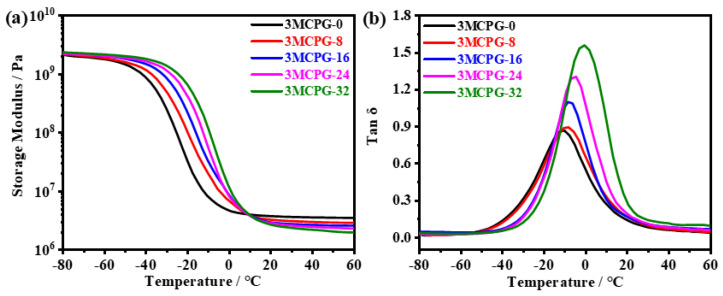
DMA curves of the MPU/AO-80 composites: variation in the (**a**) storage modulus and (**b**) loss factor as a function of temperature.

The dynamic mechanical properties of the MPU/AO-80 composites were subsequently investigated. As shown in Figure 10, increasing the amount of AO-80 did not remarkably alter the storage modulus of the material in the glass state in relation to that of the matrix, because the rigidity of AO-80 is similar to that of the matrix; that is, the ability of the composite to store elastic deformation energy is not significantly improved with the addition of the AO-80. When the temperature is increased to approximately 10 °C, the material is in the rubber state, and the storage modulus of the MPU/AO-80 composite becomes lower than that of the pristine MPU matrix. As the amount of AO-80 increases, the difference between the flexibility of the two components increases because AO-80 begins to soften with the increasing temperature and functions as a plasticizer.

As shown in Table 3, the *T*_g_ of the MPU/AO-80 composites increased with increasing AO-80 content, which is consistent with the DSC results. The tan *δ*_Max_ value and the integral area of the region with tan *δ* ≥ 0.3 also increased gradually, because AO-80 formed strong sacrificial hydrogen bonds with the polar groups of the MPU matrix. Under the action and control of periodic mechanical stress, these sacrificial hydrogen bonds can easily break and re-form, thereby increasing energy dissipation. The 3MCPG-based MPU possesses a favorable damping property, owing to the presence of side-chain methyl groups [33]. In addition, according to the DSC results, the mobility of the molecular chains increased with an increase in the AO-80 content. Thus, when the material absorbs external energy, the friction between its molecular chains increases, and its damping property is significantly improved. The tan *δ*_Max_ of 3MCPG-32 is as high as 1.56, which is close to that of conventional rubber-based damping materials, and the effective damping temperature range (tan *δ* ≥ 0.3) is 40.7 °C.

## 3. Materials and Methods

### 3.1. Materials

3MCPG (*M*_n_ = 1400 g/mol) was purchased from INVISTA Co., Ltd. (Victoria, TX, USA). MDI was purchased from Acros. TME was purchased from Sigma Aldrich. AO-80 was purchased from Asahi Denka Co., Ltd. (Tokyo, Japan). Stearic acid was purchased from Fengyi Oil Technology Co., Ltd. (Shanghai, China). Dibenzothiazole disulfide and 2-mercaptobenzothiazole were purchased from Kemai Chemical Co., Ltd. (Tianjin, China). Sulfur was purchased from Deli Chemical Co., Ltd. (Guangzhou, China). All the raw materials were used as received, without any treatment.

### 3.2. Preparation of MPU and MPU/AO-80 Composites

**Scheme 1:**
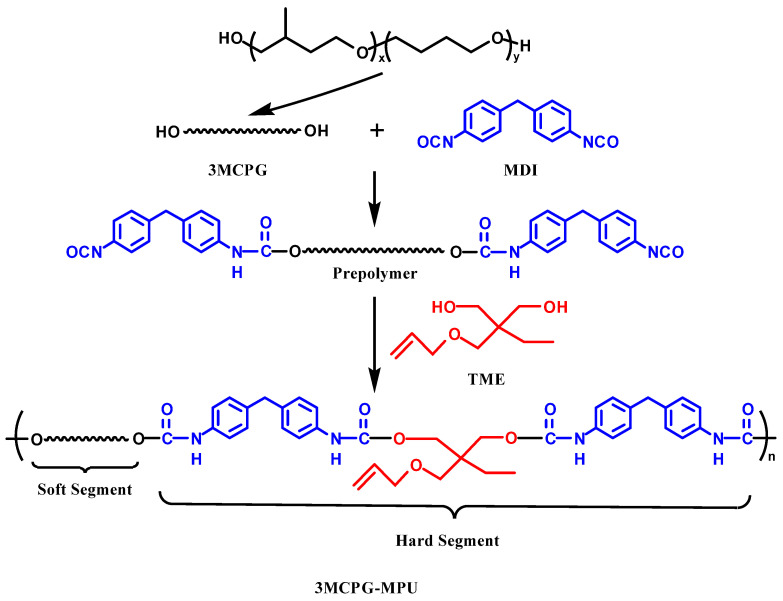
Synthetic route of the 3MCPG-based MPU.

3MCPG-based MPU was prepared from 3MCPG, MDI, and TME. Scheme 1 shows the synthesis route of the polyurethane. The molar ratio of –OH groups in 3MCPG and TME to –NCOs group in MDI was 1:1. We weighed 100 g of 3MCPG, 35.75 g of MDI and 12.45 g of TME, respectively. First, metered 3MCPG was dewatered at 115 °C under vacuum for 1.5 h. Next, metered MDI was added to the dewatered 3MCPG, and the prepolymer was prepared by reacting at 75 °C for 30 min. Finally, metered TME was added to the prepolymer, and the mixture was reacted for approximately 30 min. The samples were poured into a mold, placed in a 90 °C oven, and then cured for 10 h.

To prepare the MPU/AO-80 composites, an open mill was used to evenly mix raw PU rubber with sulfur, other vulcanizing agents, and AO-80. The detailed composition of the composites and the sample codes are summarized in Table 4. The composites are denoted as 3MCPG-X, where X represents the amount (phr) of AO-80 added to the composite.

### 3.3. Characterization

The Fourier transform infrared (FTIR) spectrum of raw MPU rubber was recorded in the attenuated total reflectance (ATR) mode in the wavenumber range of 600–4000 cm^−1^ at a resolution of 4 cm^−1^ (Tensor 27, Bruker, Karlsruhe, Germany). The as-synthesized raw MPU rubber was cut into rectangular specimens (2 cm × 2 cm × 0.5 cm), ensuring that the sample surface was flat and clean; 32 scans were recorded to obtain a spectrum.

The number of double bonds in raw MPU rubber was determined by ^1^H NMR spectroscopy (V600, Bruker, Karlsruhe, Germany) conducted using CDCl_3_ as the solvent.

The brittle cross-sections of the MPU/AO-80 composites were observed through scanning electron microscopy (SEM; S-4800, Hitachi Ltd., Tokyo, Japan). The crosslinking process of the MPU/AO-80 composites was evaluated by axial preheating at 150 °C using a vulcanizer (MR-C3, High-Speed Rail Testing Instruments Co., China).

The crosslinking density of the MPU/AO-80 composites was studied using an NMR crosslinking densitometer (VTMR20-010V-1, Suzhou Niumag Corporation, Suzhou, China) at a frequency of 15 MHz, magnetic induction strength of 0.5 ± 0.05 T, and temperature of 90 °C.

Differential scanning calorimetry (DSC) of the MPU/AO-80 composites was performed using a STARe System DSC system (Mettler-Toledo International, Inc., Switzerland). The test sample was heated from −100 to 160 °C at the rate of 10 °C/min under a nitrogen flow (50 mL/min).

Thermogravimetric analysis (TGA) of the MPU/AO-80 composites was performed using a STARe TGA System (Mettler-Toledo International, Inc., Greifensee, Switzerland) by heating the test sample from room temperature to 600 °C in a nitrogen atmosphere at a heating rate of 10 °C/min. Small specimens cut from vulcanized MPU/AO-80 composites were used for DSC and TGA.

The mechanical properties of the MPU/AO-80 composites were evaluated at a strain rate of 200 mm/min using a tensile machine (Txet Port II, Zwick/Roell, Ulm, Germany). Dumbbell-shaped specimens with 75 mm length, 4 mm width and 1 mm thickness were used.

Further, the dynamic mechanical analysis (DMA) of the MPU/AO-80 composites was conducted using a dynamic mechanical analyzer (DMA; Q800, TA Instruments, New Castle, DE, USA) over the temperate range of −80 to 80 °C at a heating rate of 3 °C/min. The frequency and strain used for DMA were 10 Hz and 0.1%, respectively. The size of the test sample was 30 mm × 4 mm × 1 mm.

## 4. Conclusions

In this work, MPU samples were synthesized from 3MCPG, MDI, and the unsaturated chain extender TME, after which MPU/AO-80 composites were prepared. The damping temperature range of the composites could easily be adjusted by controlling the amount of AO-80 added to the composites. The tan *δ*_Max_ of the composites increased by 81% from 0.86 to 1.56, and their damping property was enhanced when AO-80 was added at 32 phr. The formation of hydrogen bonds between the MPU and AO-80 improved the mechanical and damping properties of the composite. This study provides a novel strategy for the design and preparation of new high-performance PU-based damping composites and promotes the development of vibration- and noise-reduction materials for industrial applications and everyday life.

## Figures and Tables

**Table 1:** Parameters of the Kissinger equation for 3MCPG-0 and 3MCPG-32.

Samples	Heating Rate *β* (K/min)	Peak Temperature *T*_p_ (°C)	Peak Temperature *T*_p_ (K)	1/*T*_p_	ln(*β*/*T*_p_^2^)
3MCPG-0	10	186.67	459.82	0.002175	−9.9425
	15	194.50	467.65	0.002138	−9.5644
	20	198.33	471.48	0.002121	−9.2967
	25	202.08	475.23	0.002104	−9.0830
3MCPG-32	10	182.88	456.03	0.002193	−9.9591
	15	189.15	462.30	0.002163	−9.5874
	20	193.80	466.95	0.002142	−9.3160
	25	196.00	469.15	0.002132	−9.1087

**Table 2:** TGA data of the MPU/AO-80 composites.

Sample	Weight Loss (%)		Residual Mass (%)	Initial Decomposition Temperature (*T*_5%_; °C)
	Phase 1	Phase 2		
3MCPG-0	65.8	31.6	2.6	267.0
3MCPG-8	65.7	31.6	2.7	270.0
3MCPG-16	64.5	32.7	2.8	271.8
3MCPG-24	64.1	33.0	2.9	275.0
3MCPG-32	63.7	33.4	2.9	278.5

**Table 3:** Mechanical properties of the MPU/AO-80 composites, as evaluated by DMA.

Samples	*T*_g_/°C	tan *δ*_Max_	tan *δ* ≥ 0.3			*S*_tan *δ*≥0.3_
			*T*_1_/°C	*T*_2_/°C	Δ*T*/°C	
3MCPG-0	−10.3	0.86	−29.4	6.7	36.1	11.35
3MCPG-8	−8.5	0.89	−27.5	11.0	38.5	12.95
3MCPG-16	−8.3	1.09	−25.2	10.1	35.3	14.59
3MCPG-24	−4.6	1.30	−24.4	12.3	36.7	19.43
3MCPG-32	−0.6	1.56	−22.6	18.1	40.7	26.70

**Table 4:** Formulations of the MPU/AO-80 composites.

Samples	HS Content (%)	MPU (phr)	Stearic Acid (phr)	Dibenzothiazole Disulfide-Zinc Chloride Complex (phr)	Dibenzothiazole Disulfide (phr)	2-Mercaptobenzothiazole (phr)	Sulfur (phr)	AO-80 (phr)
3MCPG-0	33	100	0.5	1	3	1.5	1.5	0
3MCPG-8	33	100	0.5	1	3	1.5	1.5	8
3MCPG-16	33	100	0.5	1	3	1.5	1.5	16
3MCPG-24	33	100	0.5	1	3	1.5	1.5	24
3MCPG-32	33	100	0.5	1	3	1.5	1.5	32

## Data Availability

Not applicable.
